# The Transitivity Index: Using Transitivity as a continuous measure to account for clitic case alternation in Spanish causative constructions

**DOI:** 10.1371/journal.pone.0246834

**Published:** 2021-02-25

**Authors:** Gustavo Guajardo

**Affiliations:** Department of Language and Culture, AcqVA Aurora Centre, UiT The Arctic University of Norway, Tromso, Norway; University of California Santa Barbara, UNITED STATES

## Abstract

In Spanish causative constructions with *dejar* ‘let’ and *hacer* ‘make’ the subject of the embedded infinitive verb can appear in the accusative or the dative case. This case alternation has been accounted for by resorting to the notion of direct vs. indirect causation. Under this account, the accusative clitic with a transitive verb denotes direct causation while the dative clitic with an intransitive verb expresses indirect causation. The problem with this account is that we lack an independent definition of (in)direct causation in this context and so this approach suffers from circularity: the case of the clitic is used to determine causation type and causation type implies use of one or the other grammatical case. Therefore, a more objective way to account for clitic case alternation is needed. In this paper, I offer one possible solution in this direction by investigating clitic case alternation against Hopper and Thompson’s Transitivity parameters and a small number of other linguistic variables. The novelty of this approach is that I operationalise Transitivity as a weighted continuous measure (which I call the Transitivity Index) and use it to predict the case of the clitic. The results indicate that the transitivity of the infinitive verb, the animacy of the object and the agentivity of the subject are strong predictors of clitic case. Moreover, the Transitivity Index clearly shows that higher levels of Transitivity are associated with the dative clitic contrary to other contexts in which accusative is said to be more transitive. The findings in this paper allow us to arrive at a finer-grained characterization of the contexts in which each clitic case is more likely to occur and provide further evidence of the pervasiveness of Transitivity in natural language.

## 1 Introduction

Spanish lacks overt case marking on noun phrases (NPs), but the pronominal system still shows some vestiges of case marking. This is clearly the case with third-person pronominal elements, which distinguish between nominative (1a), accusative (1b) and dative cases (1c). I refer to these elements as pronominals (as opposed to pronouns) because, technically speaking, the nominative set comprises strong pronouns whereas the accusative and dative sets are made up of pronominal clitics. Spanish clitics differ from strong pronouns in that they cannot be conjoined, stressed or used contrastively (for an extensive review of Spanish clitics see [1]). Note that examples without reference to their source are my own.

1a.    **Él**    corrió.

he    ran.3s.

‘He ran’

1b.    Ella    **lo**    vio.

she    him.acc    saw.3s

‘She saw him’

1c.    Ella    **le**    gritó.

she    him.dat    screamed.3s

‘She yelled at him’

Generally speaking, nominative marks the subject of the sentence, accusative the direct object of a transitive verb and dative case is used for the indirect object. However, this one-to-one mapping between grammatical function and case marking does not always obtain. For example, with reverse-psychological predicates such as *asustar* ‘to frighten’ or *molestar* ‘to bother’ the experiencer argument can appear in either the accusative (2a) or the dative case (2b).

2.    a.    Las    víboras    **lo**    asustan.

the.fem.pl    snakes    him.acc    frighten.3pl

‘Snakes scare him.’

b.    **Le**    asustan    las    víboras.

him.dat    frighten.3pl    the.fem.pl    spiders

‘Snakes scare him.’

Several analyses have attempted to characterize and account for the case alternation exhibited in (2). Some have argued that it depends on the eventuality denoted by the sentence [2] and others that Transitivity factors are key in determining the case of the clitic [3–6]. The findings suggest that accusative marking is more likely with high Transitivity contexts such as agentive subjects, telic predicates and affected objects. In contrast, dative marking is most likely found with stative and atelic predicates, non-agentive subjects and non-affected objects.

Another construction where the clitic case alternation is found is with the causative predicates *dejar* ‘to let’ (3) and *hacer* ‘make’ (4).

3.    **Lo/ le**    dejan    correr    afuera.

him.acc/ him.dat    let.3pl.pres    run.inf    outside

‘They let him run outside.’

4.    **La/ le**    hacen    caminar    mucho.

her.acc/ her.dat    make.3pl    walk.inf    much

‘They make her walk a lot.’

The case alternation in this construction has attracted a lot of attention in the literature. The first accounts argued that the alternation could be explained by whether the infinitive verb was transitive or intransitive; transitive verbs require dative marking and intransitive verbs accusative case [7, 8]. This pattern was not only found in Spanish, but it is a more general characteristic cross-linguistically [9]. Despite the appeal for its simplicity and cross-linguistic coverage, this account cannot capture some data in Spanish where the opposite case appears to the one that would be expected. That is to say, it is not uncommon in spontaneous production to find examples of transitive verbs with accusative clitics (5) and intransitive verbs with dative clitics (6) (e.g., [10] and references therein). Note that the name of the country in the examples indicate the country where the sentence is found in the corpus; the number indicates the ID in the dataset.

5.    Los    bancos    no    lo    dejaban    resolver

the.masc.pl    banks not    him.acc    let.3pl.past    solve.inf

la    crisis

the.fem.sg    crisis

‘The banks wouldn’t let him solve the crisis’

(Colombia: 4380)

6.    La    tos    no    le    deja    dormir

the.fem.sg    cough not    him.dat    let.3s    sleep.inf

‘The cough doesn’t let him sleep.’

(Mexico: 1536)

In (5) the infinitive verb *resolver* ‘to solve’ is transitive so the dative clitic *le* is expected but instead we find the accusative *lo*. In (6) the infinitive verb is *dormir* ‘to sleep’, a prototypical intransitive verb, and yet the clitic appears in the dative form instead of the expected accusative case. To account for this type of data, researchers have resorted to the semantic notion of (in)direct causation. Under this account, a transitive predicate with an accusative clitic expresses direct causation while an intransitive predicate with a dative clitic denotes indirect causation [10, 11]. This account does not say anything about the type of causation implied when the case of the clitic matches the expected value (i.e., transitive → dative and intransitive → accusative). One issue with this explanation is that it suffers from circularity. That is to say, the case of the clitic is used to determine the type of causation expressed in the sentence and then the claim is that the difference in the grammatical case of the clitic expresses a difference in causation. The reason for this circularity lies in the fact that causation type has not been independently defined to act as a diagnostic for, and explanation of, clitic case. This becomes apparent when nothing can be said about causation type when the clitic matches the expected case. If causation type were an independently defined concept, we should be able to characterize each and every context where causation is called for not only those cases that are ‘exceptions’.

This paper is an attempt to offer a more systematic and objective way to account for the case alternation of clitics in causative constructions. Adopting Hopper and Thompson’s Transitivity parameters [12] together with other linguistic variables such as tense, Country and causative type, I analyse a dataset of 4,589 sentences. The analysis is conducted within a Bayesian inference framework by means of a mixed-effects logistic regression model that was fit in two different ways: in Model-1 the Transitivity parameters are entered individually as binary categorical variables whereas in Model-2 the parameters are quantified such that a unique Transitivity Index is computed for each sentence. The Transitivity Index is then used as the main predictor in Model-2 (see details in the Methodology section).

### 1.1 The Transitivity parameters

On the basis of cross-linguistic evidence, Hopper and Thompson [12] propose that transitivity should be construed as a scale that applies at the clause level as opposed to being a property of just the verb. In their view, Transitivity is composed of ten parameters addressing features of the subject, verb and object of the clause as shown in Table 1. All the parameters are binary except for Individuation, which describes features of the object and is made up of the six sub-parameters in Table 2. For expository purposes I use *Transitivity* with an upper-case *T* to refer to the global property of a clause and *transitivity* with a lower-case *t* to refer to the property of transitive verbs.

**Table 1:** Hopper and Thompson’s Transitivity parameters.

Component	High	Low
PARTICIPANTS	2 or more	1
KINESIS	action	non-action
ASPECT	telic	atelic
PUNCTUALITY	punctual	non-punctual
VOLITIONALITY	volitional	non-volitional
AFFIRMATION	affirmative	negative
MODE	realis	irrealis
AGENCY	A high in potency	A low in potency
AFFECTEDNESS OF O	totally affected	not affected
INDIVIDUATION OF O	highly individuated	non-individuated

**Table 2:** The subparameters comprising Individuation.

Individuated	Non-Individuated
Proper	common
human, animate	inanimate
Concrete	abstract
Singular	plural
Count	mass
referential, definite	non-referential

As the tables show, each parameter has a value that corresponds to higher Transitivity and the opposite value that corresponds to lower Transitivity. For example, for the Participants parameter, a transitive verb (2 participants) is higher in Transitivity than an intransitive verb with only 1 participant. The appeal of this approach is that clauses can be categorized in a scale as more or less transitive instead of relying on a categorical distinction solely based on the transitivity status of the verb. As one reviewer correctly points out, some of the parameters seem to be more gradient, or of a less binary nature, than others such as the distinction between *proper* vs. *common nouns*. While this is a valid observation, for the purpose of the statistical analysis I assume these properties to be binary while keeping in mind that the semantics of these features may be less categorical than one would like them to be. The goal of this study is to model Hopper and Thompson’s proposal as it was first proposed to assess its validity. Further improvements to the proposal can then be proposed based on these types of observations.

Transitivity can be seen at play in a variety of languages across different linguistic phenomena. For example, in the language Yukulta irrealis clauses mark the object with oblique case instead of the usual absolutive case in realis clauses [13]. In Estonian, partitive case is used instead of the accusative and genitive cases to mark the partial degree of affectedness of the object [14]. In English, Transitivity has been used to account for properties of implicit objects (e.g., John cooked [∅] this morning) distinguishing between indefinite and definite readings of this construction [15] (for a detailed account of Transitivity cross-linguistically see [12]).

In Spanish, Transitivity has been useful in accounting for differential object marking [16], non-anaphoric uses of the clitic *se* [17], inalienable possessive constructions [18] and reverse-psychological predicates [3, 6], among others.

There has been some previous work using features similar to the Transitivity parameters in the study of clitic case alternation with causatives, but to the best of my knowledge, this is the first article using a combination of statistical models and the Transitivity parameters to account for clitic case alternation in causative constructions. Enghels [11] studies the case alternation of clitics in the causative constructions with *dejar* and *hacer* in Peninsular Spanish with corpus data. She analyses 500 sentences with a number of linguistic variables such as causative (*dejar* ‘let’ or *hacer* ‘make’), dynamicity of the object and subject (animate, dynamic inanimate, non-dynamic inanimate) and the type of infinitive verb (transitive, unergative and unaccusative). She finds several differences between the realization of dative and accusative case. For example, it is reported that in general both causatives appear much more with the dative than with the accusative case and animate objects also tend to favour the dative clitic. On the other hand, the chances of finding the dative case drop as the dynamicity of the infinitival complement increases (i.e., the more dynamic the predicate, the less likely it is to find the dative clitic). With respect to the dynamic aspect of the subject, she finds an interesting dichotomy between the two causatives; very dynamic subjects with non-dynamic objects favour the accusative case with *hacer* but dynamic subjects with *dejar* are found with the dative clitic. In addition, her data show that the more dynamic objects also favour the dative clitic while abstract inanimate objects are more often found with the accusative.

An important difference between the present study and the study described above is that Enghels [11] only studied Peninsular Spanish. Studying clitic case alternation in Peninsular Spanish is problematic because *leísmo*, the phenomenon where the dative clitic *le* is used for masculine animate *direct* objects, is prevalent in this variety [19–21]. This phenomenon makes it difficult to determine the case of the clitic because the realization of the clitic as *le* cannot be unambiguously interpreted as signalling dative case when the referent is animate and masculine. In general, and particularly in non-contact varieties, Latin American Spanish, on the other hand, lacks l*eísmo* [22]. In their study on the development of *leísmo* in Spain and Latin America spanning ten centuries, Parodi et al. show that this phenomenon has been completely absent in Latin America since the 20^th^ century, with the exception of bilingual contexts where Spanish co-exists with another language such as rural areas of the Andean region, Paraguay and Ecuador (for similar observations and conclusions see [23, 24]). Based on these observations, Peninsular Spanish is not included in the present study but all other Latin American dialects are with the caveat perhaps that there might be a few cases, if any, of *leísmo* but these should not affect the overall results.

Despite the limitations of Enghels’s study, we can use her results to make very precise and testable predictions that can be evaluated in our models.

### 1.2 Hypotheses and predictions

Based on the results of Enghels’s study and Hopper and Thompson’s parameters, the following hypotheses and predictions were tested.

**Hypothesis 1:** The Transitivity parameters will co-vary in the same direction
**Hypothesis 2:** The two causative predicates will show different preferences in clitic case.
**Hypothesis 3:** Accusative case will align with higher Transitivity and dative case with lower Transitivity.

Hypothesis 1 falls out from Hopper and Thompson’s proposal that the parameters should co-vary towards the same end of the scale. This means, for example, that if a language makes a distinction between telic and atelic predicates and between definite and indefinite objects, then they predict that telic predicates should co-occur with definite objects and atelic ones with indefinite objects. Hypothesis 2 follows from Enghels’s work where she finds that *hacer* appears with the dative case more often than *dejar*. If this is a general characteristic of the construction, then we predict that the Bayes factor for the variable Causative will show positive evidence in favour of this hypothesis and the posterior mean estimate will be positive (because *Accusative* and *dejar* are the reference levels). Hypothesis 3 follows from previous work both on causatives and reverse-psychological predicates where accusative was found to occur in higher transitivity contexts [3, 6, 25]. If accusative is associated with higher transitivity, then we expect that as transitivity increases, the probability of the accusative clitic will increase and that of the dative clitic will decrease. Model-2 will allow us to test this prediction.

## 2. Methodology

### 2.1 Data and variable coding

The data were extracted from Corpus del Español [26], specifically from the Web Dialects and NOW (News on the Web) versions. The web interface of the corpus only allows for extraction of a maximum of 500 random concordances per search, thus 500 sentence fragments for each combination of causative+clitic followed by an infinitive were extracted from the Web Dialects corpus (e.g., 500 sentence fragments with *hacer* and the singular masculine clitic and 500 sentence fragments with *hacer* and the plural masculine clitic). As the dative clitic only inflects for number but not for gender this resulted in twice as many accusative clitics than dative clitics. In order to have a more balanced dataset, 2000 more sentences with the dative clitic were extracted from the NOW corpus (500 for each causative+clitic number combination). Both versions of the corpus are made up of texts from the Internet so the register is relatively similar in both; the NOW corpus contains mostly news and the Web Dialects contains language from news sources, general websites and blogs (but the data is only coded as general vs. blogs). After removal of the data from Spain and the USA as well as duplicates and false positives, the dataset contains a total number of 4589 clauses containing one of the causative verbs from 19 Spanish-speaking countries. Table 3 shows the counts and relative frequency by clitic and causative verb. A note of caution is in order regarding the way the source country has been determined in the corpus. As the creator of the corpus explains on the website, they used Google “Advanced Search” function to limit the search of pages by country. If the website contains a top-level domain such as.ar for Argentina or.mx for Mexico then it is a very simple task to determine the country of origin of the website. If the country domain is not available because the site used an international domain such as.com,.org, etc, then Google relies on other types of information. This includes the IP address, location information on the page, links to the page and any relevant information from Google places. Although not without problems, this system seems quite reliable as shown by dialect-oriented searches conducted on the corpus (and publicly available on the corpus’s website) to assess Google’s accuracy in determining country of origin. Since the focus of this article is not dialectal differences in the use of clitic case, the country of origin is only included in the models as a random effect to control for this type of variability.

**Table 3:** Raw counts and relative frequencies of each clitic form and causative verb in the final dataset.

Clitic	Count	Relative Frequency	Clitic	Count	Relative Frequency	Causative	Count	Relative Frequency
*lo*	602	0.13	*las*	578	0.13	*hacer*	2157	0.53
*los*	698	0.15	*le*	1051	0.23	*dejar*	2432	0.47
*la*	610	0.13	*les*	1050	0.23			

The annotation of the data was conducted manually using the Transitivity parameters as well as three additional variables. Table 4 shows all the variables used and the possible values of each. Not all of Hopper and Thompson’s parameters were considered, however. *Volitionality* being almost indistinguishable from *agency* was discarded and only *Agency* was included. For the *Individuation* parameters, *referential* was not included because most objects were referential in this construction and there were no proper nouns in the sample so *proper* vs. *common* was not included. The four additional variables included in the analysis are *causative*, *tense*, *person*, *NumberSubj*. All of these variables refer to grammatical features of the causative. Due to data sparsity (i.e., very few data points of some levels of a variable) *Tense* and *Person* were coded as binary variables. *Tense* was coded as *past* vs. *non-past* and *Person* as *3*^*rd*^ vs. *non-3*^*rd*^. *NumberSubj* refers to the number feature of the subject of the causative verb (*singular* vs. *plural*).

**Table 4:** Predictor variables and possible values of each.

Variable Name	Possible Values	Variable name	Possible Values
AFFECTEDNESS	*affected/ non-affected*	MODE	*indicative/ subjunctive*
AFFIRMATION	*affirmative/ non-affirmative*	NUMBER_OBJ	*sg/ pl*
AGENCY	*high/ low*	NUMBER_SUBJ	*sg/ pl*
ANIMATE	*animate/ inanimate*	PARTICIPANTS	*transitive/ intransitive*
ASPECT	*telic/ atelic*	PERSON	*3rd/ non-3rd*
CAUSATIVE	*dejar/ hacer*	PUNCTUALITY	*punctual/ non-punctual*
CONCRETENESS	*concrete/ abstract*	TENSE	*past/ non-past*
COUNT	*count/mass*	TRANSITIVITY INDEX	*continuous between 0–1*
KINESIS	*state/ non-state*		

### 2.2 Statistical analysis

The statistical analysis was conducted in R version 4.0.3 [27]. The dataset was first randomly partitioned into three smaller subsets with the caret package [28]. Two subsets contained 20% of the data and the remaining 60% of the data comprised the third subset. This means that each of the smaller datasets contained 918, 917 and 2754 sentences, respectively. The first dataset with 918 sentences was used to calculate the weight of each parameter. The dataset with 60% of the data was used to fit two Bayesian generalized mixed-effects logistic regression models and the dataset with the remaining 20% of the data was used to test the predictive power of these models.

#### 2.2.1 The Transitivity Index

The Transitivity Index was calculated by training 1000 random forests with 3000 trees each with the party package [29]. The conditional variable importance measure [30] was then calculated for each of the 1000 random forests with the permimp package [31].

Variable importance is a measure calculated by randomly permuting a predictor, thus breaking the original association of the predictor with the response variable. The difference in prediction accuracy before and after the permutation averaged over all trees in a random forest is the variable importance. Intuitively, if there is a strong association between the predictor and the response variable, the prediction accuracy will be severely affected after the permutation. On the other hand, if the predictor variable is not predictive of the response variable, then prediction accuracy should remain unaffected (or should, at least, not decrease substantially). There are several ways in which the random permutation can be performed, the variable importance adopted here is one of the most robust and reliable types as it is designed to avoid bias toward correlated predictor variables [30, 32]. The higher the variable importance score, the more important the predictor is. The final weight for each parameter was the result of averaging over the 1000 individual variable importance measures of each random forest.

The procedure was repeated twice to confirm the results were reliable. There was a perfect correlation of 1 between the two sets of averaged weights. The weights for each parameter are shown in Table 5 in decreasing order of importance. This means that participants, that is whether the main verb is transitive or intransitive, is the most important variable, followed by agency of the subject and animacy of the object. The three least important variables are telicity, number of the object and affirmation. The parameter Individuation was calculated by adding up the weights for each of the four subparameters animacy of object, concreteness, count and number of object. Assignment of the weights to each parameter in each sentence was done such that if the parameter had the higher value for Transitivity it was assigned the numerical weight, otherwise it was assigned 0. For example, for the animacy of object parameter a sentence was assigned 0.004709 if the object was animate and 0 if the object was inanimate. The total Transitivity Index for each sentence was then calculated by adding up all the values of the individual parameters by sentence. To ease interpretation, the final index was normalized between 0 and 1.

**Table 5:** Average weights for each Transitivity parameter.

Parameter	Weight	Parameter	Weight	Parameter	Weight
Participants	0.121932	Mood	0.000160	Punctuality	-0.000063
Agency of subject	0.006757	Affectedness	0.000135	Telicity	-0.000216
Animacy of object	0.004709	Concreteness	0.000110	Number of Object	-0.000500
Kinesis	0.002466	Count	0.000008	Affirmation	-0.000638

This method was chosen in order to assign different weights to the parameters to reflect the fact that not all parameters are likely to have the same level of importance (i.e., weight) in every construction. For example, one can imagine that individuation may be an important parameter in one construction but much less relevant in a different construction within the same language. Likewise, cross-linguistically a parameter may be more or less important in the same construction or phenomenon depending on the language (e.g., *animacy* vs. *specificity* in differential object marking).

#### 2.2.2 The mixed-effects logistic regression models

Two Bayesian mixed-effects logistic regression models were fitted with the Stan modelling language [33] in the brms package [34]. For Model-1, four sampling chains ran for 8000 iterations each with a warm-up period of 4000 iterations. Model-2 uses 4000 iterations each with a warm-up period of 2000. The difference in the number of iterations between the two models is due to the complexity of each model. Since Model-1 contains far more parameters and a different random effects structure, it needed more iterations for the chains to mix well. I followed the recommendations in Gelman et al. [35] for the choice of prior distributions. For the fixed effects, I used a Cauchy weakly informative prior distribution with centre 0 and scale 2.5 (0, 2.5) and the intercept has a scale of 10 (0,10). This Cauchy distribution prior on the fixed effects gives preference to values less than 5 but it also allows for the possibility (25%) of very large values should the data show evidence for this [35]. For the prior distribution on the random effects, I used the default setting in the brms package, namely a Student’s *t-*distribution (*v =* 3, *μ* = 0, *σ* = 10).

Model-1 is a Bayesian mixed-effects logistic regression fitted with the Transitivity parameters individually such that each parameter can contribute separately to the model. In addition, four extra variables causative, number of subj, person and tense were included in the model. The model was fitted with a number of interactions based on the findings from the literature discussed above, namely AgencySubj*AnimacyObj, Participants*Causative, Participants*AgencySubj, AgensySubj*Causative, Concreteness*Participants, Count*Participants. The effect of each predictor variable was tested by means of Bayes factors. A null region was first calculated with the bayestestR package [36]. A null region is an interval that is practically equivalent to 0. This means that if the posterior distribution of a predictor falls within this region, we cannot assert that there is evidence against the null hypothesis. The Bayes factor computes the posterior odds of the posterior probability within the null region and the posterior probability outside the null region. The interpretation of Bayes factors is as follows [37]: BF < 1 evidence in favour of the null hypothesis (i.e., the parameter does not contribute to explaining the outcome), BF = 3–10 there is moderate evidence, BF = 10–30 there is strong evidence, BF = 30–100 there is very strong evidence and BF > 100 extreme evidence.

Model-2 is also a Bayesian mixed-effects logistic regression but the main predictor variable is Transitivity Index. The other four variables were also included in the model so that both models could be compared on the same predictor variables.

Besides looking at the posterior distributions of the models to study the evidence in favour or against the effect of each predictor variable, I also analyse and compare the predictive power of Model-1 and Model-2 to determine whether the Transitivity Index is a reliable measure of transitivity in comparison with the individual parameters.

Model selection was performed by comparing models with different random effect structures, ranging from intercept-only models to more complex random intercept and slope models. Model comparison was conducted by comparing the predictive power of each model by means of model stacking and LOO (leave one out) cross-validation [38] with the loo package [39]. Both methods yielded the same results and chose the same model as the best model. All the fixed parameters were included in the final model because the goal of the analysis is not to find the best model but to evaluate the effect of each of the Transitivity parameters on the case of the clitic as well as compare the predictive performance of Model-1 to that of Model-2. Since Model-2 contains all the parameters in the form of the index, it seems methodologically more appropriate to keep all parameters in Model-1 as well.

## 3. Results

### Model-1

Model-1 contains ten single terms and six interaction terms. The single terms are Affirmation, affectedness, telicity, tense, punctuality, person, NumbObj, mood, kinesis and NumberSubj. The interactions are AgencySubj*AnimacyObj, participants*causative, participants*agencySubj, agencySubj*causative, concreteness*participants and count*participants. In addition, the model contains country as a random intercept and random slopes for Participants, AgencySubj and AnimacyObj by Verb.

I will first present the results of the Bayes factor analysis that shows which parameters offer substantial evidence in explaining the dependent variable (ie., clitic case). The Bayes factors are shown in Table 6. For an effect to offer at least moderate evidence for its importance, the Bayes factor should at least be 3. This means that none of the predictors that do not participate in interactions (i.e., affirmation, affectedness, telicity, tense, punctuality, person, NumbObj, mood, kinesis and NumberSubj) make a significant contribution to explaining the case of the clitic. The parameters for which there is significant evidence to reject the null hypothesis are AgencySubj, AnimacyObj, causative, concreteness and participants. Count is not deemed important either in the interaction with Participants or as a single term.

**Table 6:** Bayes factor results indicating the posterior log odds against the null hypothesis of no effect of each predictor.

Parameter	BF	Parameter	BF	Parameter	BF
AgencySubj	3443.61	Affirmation	1.05	Telicity	0.08
Causative	97.01	AgencySubj*Participants	1.00	Punctuality	0.06
AnimacyObj	91.52	Participants*Count	0.40	Kinesis	0.04
AgencySubj*AnimacyObj	36.38	Person	0.39	Mood	0.01
Concreteness	14.55	Affectedness	0.18	Tense	0.01
Participants	5.66	Causative*AgencySubj	0.16	NumberObj	0.01
Participants*Concreteness	3.07	Count	0.13		
NumberSubj	1.62	Causative*Participants	0.09		

The larger the Bayes factor, the more evidence against the null hypothesis. The shaded areas show Bayes factors larger than 3.

For ease of exposition, I present the results of the model in two formats. First, I show the posterior distribution intervals of the terms for which there is enough evidence that they contribute to explaining the case of the clitic according to the Bayes factors. The exception to this is the interaction Participants*Count, which must be calculated because even though the interaction *per se* is not very informative, Participants is relevant in other interactions and so I cannot remove the interaction from the calculation. Second, I will present the results of the interactions via marginal effects plots because they offer a nice and reader-friendly way to interpret interactions. A complete table of posterior coefficient estimates, standard errors, 95% credible intervals and convergence diagnostics of Model-1 can be found in the S1 Appendix.

**Fig 1:**
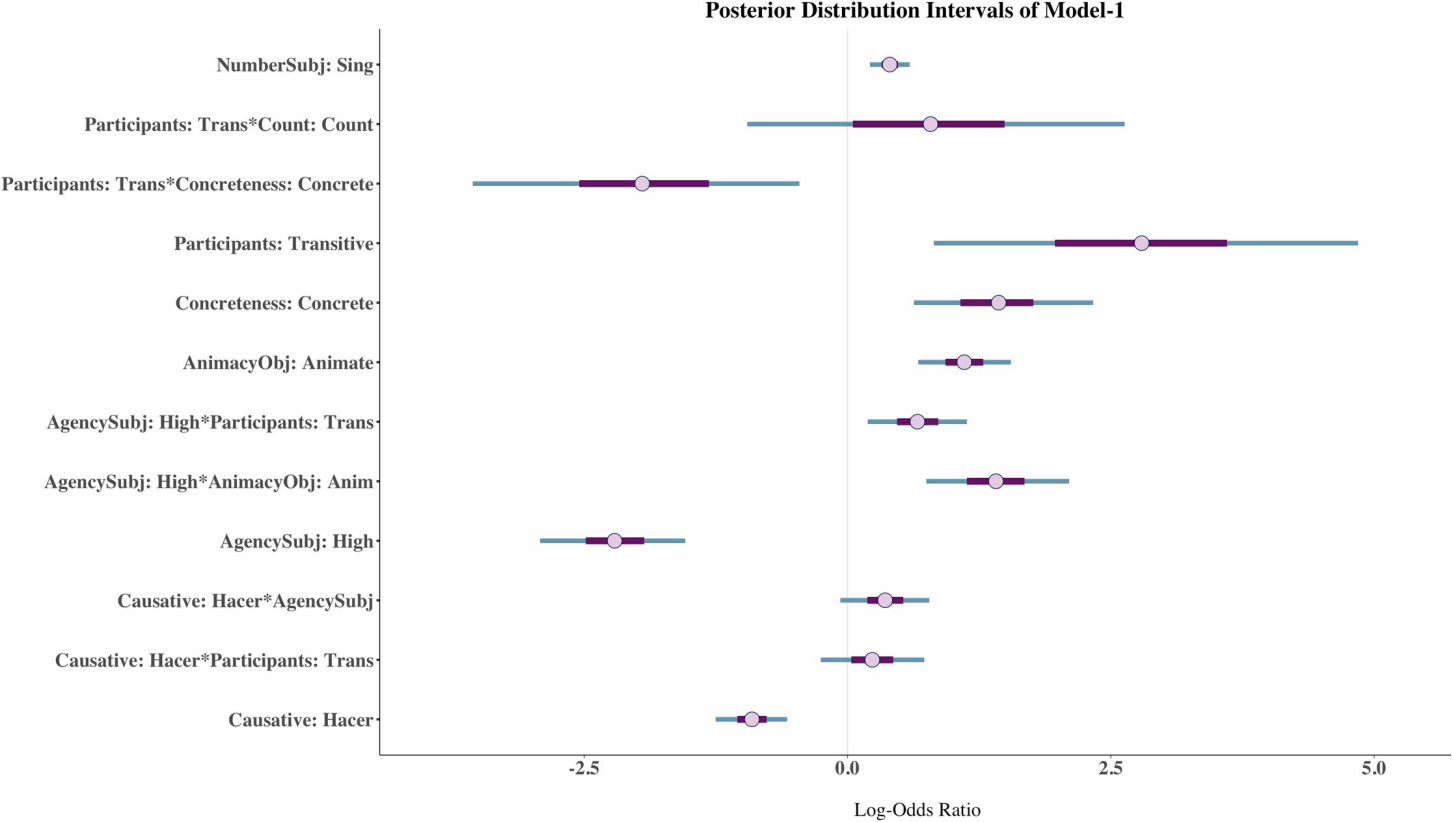
Posterior distribution intervals of terms with a Bayes factor larger than 1 in Model-1. The thicker purple lines show 50% and the thinner teal lines 90% credible intervals. The dot represents the posterior mean estimate.

Fig 1 shows the posterior distribution intervals of all terms whose Bayes factor is larger than 3. The posterior distribution intervals allow us to see the degree of uncertainty of the posterior estimate. The smaller the credible interval, the more certain we can be that the coefficient estimate lies within that interval. The posterior distribution intervals in Fig 1 show quite a high degree of certainty as they are rather small with three exceptions. The first exception is the interaction Participants*Count, whose posterior mean estimate is 0.79 (CI: -1.30, 3.00). We saw that the Bayes factor for this interaction was 0.40, meaning that the data is 2.5 times more probable under the null (i.e., 1/0.40). The fact that the credible interval (CI) contains zero corroborates that it is likely that this interaction has a null effect on the outcome. The interaction Participants*Concreteness also shows a relatively larger posterior distribution interval. In contrast with the previous interaction, however, the Bayes factor for this interaction is 3.07, which shows a relatively moderate degree of positive evidence for an effect. The posterior mean is -1.95 (CI: -3.92, -0.17) and we see that the CI does not contain zero, supporting the existence of a real effect. Since the posterior mean is negative, it indicates that transitive verbs with concrete objects disfavour the dative clitic (i.e., in comparison to intransitive verbs with abstract objects). The large CI (i.e., the higher degree of uncertainty) is likely due to the small number of abstract objects compared to concrete objects in the data (6% vs. 94%). The third posterior distribution interval that looks slightly wider than the rest is Participants. But, since participants is part of four interactions, this posterior coefficient estimate is the value of Participants with abstract and mass objects with the causative *dejar* “let” and non-agentive subjects. The Bayes factor for this parameter is 5.66, which shows moderate evidence against the null hypothesis. The posterior mean is 2.79 (CI: 0.43, 5.27), indicating that when *dejar* appears with a transitive verb with a non-agentive subject and an abstract mass object it favours the dative clitic.

**Fig 2:**
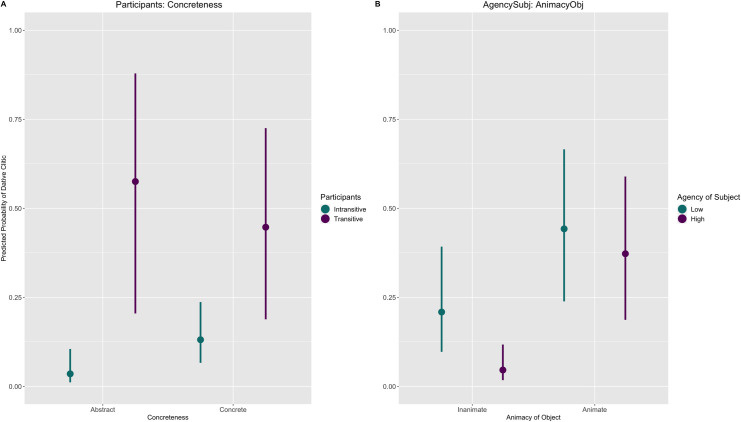
Marginal effects of Model-1. The y-axis represents the posterior predicted probability of the dative clitic.

Fig 2 shows the marginal effects of the interaction terms. The predicted estimate is the mean of all drawn posterior samples and the confidence intervals are Bayesian predictive intervals.

Plot (A) shows the interaction Participants*Concreteness. We can observe that transitive verbs show much more uncertainty than intransitive verbs, suggesting that their behaviour is less systematic compared to intransitive verbs. The interaction shows that there is a large difference in the behaviour of abstract objects according to whether the verb is transitive or intransitive. The predicted mean for the dative clitic with abstract objects and intransitive verbs is just 0.03 as in (7) whereas with transitive verbs the mean increases to nearly 0.60 as in (8). However, there are very few cases of abstract objects with transitive verbs so this is not a very strong generalization. There is also a relatively large difference with concrete nouns but the difference is much smaller than with abstract nouns (0.13 vs. 0.44).

7. […]    Jobs    tomaba    sus    ideas    y    las    hacía    parecer    propias

Jobs    take.3sg.past    his    ideas    and    them    make.3sg.past    seem.inf    own

‘Jobs would take his ideas and make them look like they were his own’

(Dominican Republic: 2012)

8.    No    pierdan    el    tiempo,    ni    lo    hagan    perder […]

not    lose.2pl.imp    the.masc.sg    time    nor    it    make.2pl.imp lose.inf

‘Don’t waste time or make others waste it’

(El Salvador: 2293)

The interaction AgencySubj*AnimacyObj is shown in Plot B. Overall, we can observe that inanimate objects disfavour the dative clitic in comparison to animate objects. The main difference in this interaction is that between subjects high in agency with animate or inanimate objects. The context that least favours the dative clitic, with a predicted mean of 0.05, is when a subject high in agency appears with an inanimate object as in (9). On the other hand, when the object is animate then the predicted mean increases to almost 0.40 as in (10).

9.    […]    echaremos    las    patatas    y    las    dejaremos

throw.1pl.fut    the.fem.pl    potatoes    and    them    let.1pl.fut

cocer […]

cook.inf

‘We will throw in the potatoes and let them cook’

(Venezuela: 415)

10.    […]    la    hizo    bajar    mediante    amenazas    con    un    cuchillo […]

her make.3sg.past    lower.inf    through    threats    with    a    knife

‘He made her get off (the bus) by threatening her with a knife’

(Argentina: 172)

### Model-2

**Fig 3:**
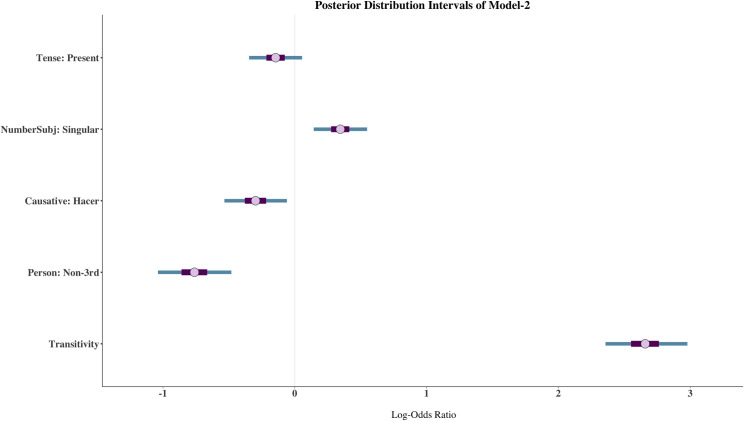
Posterior distribution intervals of Model-2. The dot represents the posterior mean estimate. The thicker purple lines show 50% and the thinner blue lines 90% credible intervals, respectively.

**Fig 4:**
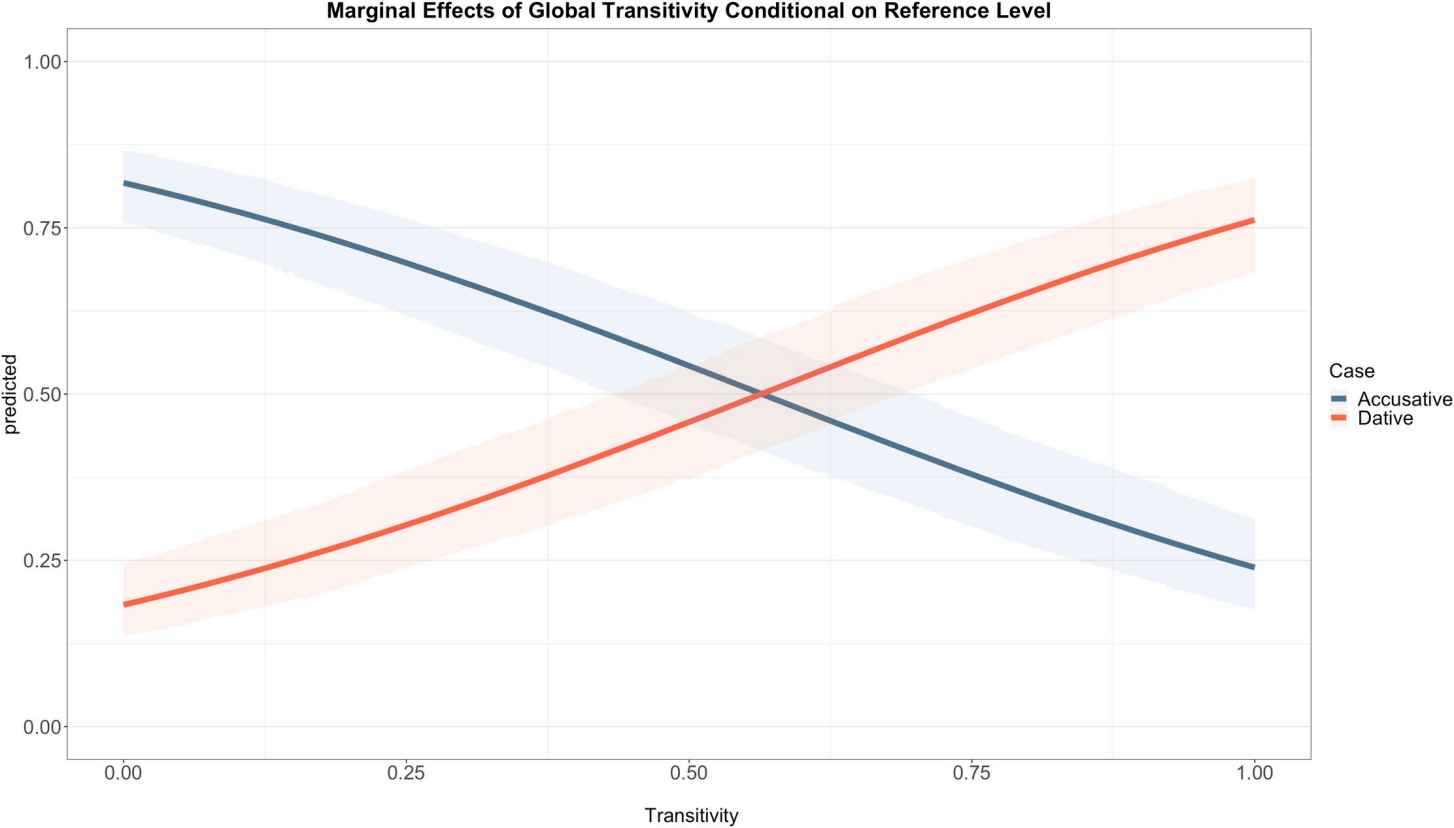
Marginal effects of the Transitivity Index conditional on the reference level of the other predictors. For Set 1 the reference levels are Person: *non-3*^*rd*^, Tense: *non-past*, Causative: *hacer*, NumberSubj: *singular* and for Set 2 Person: *3*^*rd*^, Tense: *past*, Causative: *dejar*, NumberSubj: *plural*. The y-axis represents the posterior predicted probability for the dative clitic and x-axis the Transitivity Index scaled to have standard deviation 0.5.

Model-2 comprises the Transitivity Index as the main predictor of interest and the four extra variables that were also part of Model-1. Unlike Model-1, the best model was the model with only random intercepts in verb and country. Fig 3 shows the posterior distribution intervals of the predictor variables. The posterior mean estimate of Transitivity is 2.66 (CI: 2.36, 2.98) suggesting that an increase of one unit in Transitivity increases the log-odds of the dative clitic. This can be clearly seen in Fig 4 that shows the marginal effects of Transitivity. The line colours show the posterior predicted probabilities for the accusative (blue) and dative (orange) clitics. We can observe that for the lowest level of Transitivity the accusative clitic has a predicted mean of 0.82 whereas that of the dative clitic is just 0.18. On the other end of the continuum, that is with the highest level of Transitivity, the dative clitic has a predicted mean of 0.76 while the predicted mean of the accusative clitic is 0.24. Both clitics are equally probable around the medium Transitivity range. In addition, the Bayes factor for the Transitivity Index is over 10000 demonstrating that Transitivity is an extremely strong predictor of clitic case. The only other variable that showed evidence of an effect against the null hypothesis in Model-2 was Person (BF = 4320), suggesting that a subject that is non-3^rd^ person disfavours the dative clitic as the mean estimate is -0.76 (CI: -1.04, -0.48).

### Predictive performance of Model-1 and Model-2

Table 7 shows the confusion matrices for Model-1 and Model-2. The predictive power of both models is examined on training and testing data. Note that the performance on training data tends to always be more optimistic than on testing data since the model has learned the pattern from the training data. The performance on the testing data reflects the true predictive power of the model.

**Table 7:** Confusion matrices for Model-1 and Model-2 on training and testing data.

Training Data						Testing Data					
Model 1			Model 2			Model 1			Model 2		
	Reference			Reference			Reference			Reference	
**Prediction**	Acc	Dat	**Prediction**	Acc	Dat	**Prediction**	Acc	Dat	**Prediction**	Acc	Dat
Acc	1263	345	Acc	1237	426	Acc	410	128	Acc	417	146
Dat	204	942	Dat	230	861	Dat	100	279	Dat	93	261
**Accuracy:**	**0.80**		**Accuracy:**	**0.76**		**Accuracy:**	**0.75**		**Accuracy:**	**0.74**	
95% CI:	(0.79, 0.82)		95% CI:	(0.75, 0.78)		95% CI:	(0.72, 0.78)		95% CI:	(0.71, 0.77)	
Kappa:	0.60		Kappa:	0.52		Kappa:	0.50		Kappa:	0.47	
F1:	0.77		F1:	0.72		F1:	0.71		F1:	0.69	

Shaded diagonals represent correct predictions.

The predictive accuracy on testing data is 0.75 for Model-1 and 0.74 for Model-2 so both models have almost equal predictive power. This is quite remarkable as Model-1 contains interactions that are not possible to include when the parameters are collapsed into one single numerical value. This seems to suggest that the parameter weights are able to capture the same amount of information that the single parameters with their interactions do in Model-1 so there appears to be almost no loss of information from Model-1 to Model-2. If we look at the confusion matrix, we can see that Model-1 can predict the dative clitic a bit better than Model-2. More specifically, Model-1 can predict 279 cases and Model-1 261 out of a total of 407, which translates into a relative proportion of 0.68 and 0.64, respectively. Conversely, Model-2 is slightly better at predicting the accusative clitic, as it can correctly predict 417 cases in contrast to 410 in Model-1 (i.e., 0.80 vs. 0.82).

## 4. Discussion

We will start off the discussion by first evaluating the hypotheses and predictions laid out in Section 1. I repeat the hypotheses below to make the discussion easier.

**Hypothesis 1:** The Transitivity parameters will co-vary in the same direction
**Hypothesis 2:** The two causative predicates will show different preferences in clitic case.
**Hypothesis 3:** Accusative case will align with higher Transitivity and dative case with lower Transitivity.

Hypothesis 1 is Hopper and Thompson’s original hypothesis where they claim that the Transitivity parameters will always co-vary toward one or the other end of the Transitivity scale. To determine whether this hypothesis is borne out we need to look at the coefficient estimates of Model-1. I will limit the discussion to those parameters for which there was enough evidence of an effect, namely AgencySubj, AnimacyObj, Participants and Concreteness and their interactions. For this hypothesis to be true, all estimates need to have the same sign; they all need to be negative or positive. The posterior estimate coefficients for these variables and their interactions are all positive except for AgencySubj (-2.21, CI: -3.06, -1.41) and the interaction participants*concreteness (-1.95, CI: -3.92, -0.17). Therefore, the strong version of Hypothesis 1 is not supported. Having said that, in Model-2 we saw that when Transitivity is operationalised as a continuous property we do see an increase in the predicted probabilities of the dative clitic as Transitivity increases. Thus, generally speaking, the individual parameters may not all converge on the same end of the Transitivity spectrum but overall it seems that these minor divergences may disappear when the parameters are quantified. A weaker version of the hypothesis then is supported because if it were the case that the parameters all differed in haphazard ways then we would not expect the results of Model-2.

With regard to Hypothesis 2, the prediction was that *hacer* would favour the dative clitic based on Enghels’s previous finding [11]. In Model-1 Causative participates in two separate interactions, one with AgencySubj and one with participants. The Bayes factors for these interactions were very low so we do not have evidence to support their existence. We only need to look at the mean estimate for causative. The mean posterior estimate for *hacer* with non-agentive subjects and intransitive verbs is -0.91 (CI: -1.32, -0.51), suggesting that in this context *hacer* disprefers the dative clitic. In Model-2, where no interactions were included, the posterior mean estimate is -0.30 (CI: -0.56, -0.06), confirming that *hacer* disfavours the dative clitic. These results leads us to the conclusion that Hypothesis 2 is not supported by the data.

Hypothesis 3 was formulated on the basis of findings from reverse-psychological predicates where it was found that contexts higher in Transitivity corresponded to accusative marking. It should be clear by now that this is not the case in the causative construction. Model-2 clearly shows that increasing Transitivity brings about an increase in the predicted probability of the dative, not the accusative, clitic. This is an important but not an unexpected result. There are several reasons why the dative clitic is likely to be associated with higher Transitivity in this construction that may not hold in other constructions. The first thing to remember is that, in the causative construction, transitive verbs traditionally require the subject of the infinitive to be in the dative case. Although this is not categorical, we saw in the calculation of the parameter weights that Participants is the most important variable. This is clearly seen in our data sample where 80% of transitive verbs co-occur with a dative clitic and 70% of intransitive verbs appear with an accusative clitic. In addition, the dative clitic in the causative construction appears to preferentially refer to animate objects. While 69% of the accusative-marked objects are animate and 31% inanimate, only 4% of dative-marked objects are inanimate and an overwhelming 96% are animate. This is probably due to the fact that indirect objects tend to be animate, and especially human, and not because of an intrinsic feature of the clitic itself (such as being specified with a [+animacy] feature). From a cross-linguistic perspective the association of dative objects and higher Transitivity is actually not uncommon. Hopper and Thompson [12] point out that what traditional grammars call *indirect objects* should be called Transitive Os (objects) instead of the traditional accusative objects because they tend to be definite and animate. Even in English, Givón [40] reports that out of 115 indirect objects in a text, 97% were definite and overwhelmingly animate and Hopper and Thompson themselves find that out of 33 indirect objects in one text, 100% were human.

The finding herein that the dative clitic is associated with higher Transitivity in Spanish causative constructions does not invalidate previous findings where the accusative clitic in reverse-psychological predicates has been found to signal high Transitivity. However, they do highlight the need to be cautious about drawing generalizations that go beyond the construction under study. General statements like “X property/morpheme signals higher Transitivity” should be avoided either so that they apply in the local domain of the study or until enough evidence has been amassed across different constructions. At least in the case of clitics, their behaviour seems to be highly structure-dependent, which limits our ability to reach the overarching generalizations that most linguists seek to make.

In terms of how well the models account for the data we saw that the models achieve very comparable predictive power. Interestingly, it seems that Model-2 can generalize better from the training data than Model-1 as the difference between the training and the testing accuracy is much smaller in Model-2 than in Model-1 (2% vs. 5%).

It must be acknowledged, however, that Transitivity alone cannot capture all there is in the alternation of clitic case in causative constructions. There must be other factors not included in the models that are important in the alternation. Needless to say, sociolinguistic variables such as age, gender and social class may all play a role in any type of linguistic alternation. One variable that was not included in the models is individual variation, which has been shown to be a significant factor in morphosyntactic variation [41–44]. One way to incorporate individual variation in the models could have been to use the website where the sentence fragment comes from as a proxy for speaker. Thus, models with website as a random intercept were evaluated but, because most websites appear almost only once, then this caused the models to overfit (i.e., there is almost a one-to-one correspondence between website and clitic case). In addition, we must entertain the possibility that there may be idiosyncratic factors that are simply irreducible to any one variable. Having said that, the Transitivity Index alone can account for nearly 75% of the data so whatever variable we have not accounted for is less important than Transitivity.

The Transitivity Index has great potential for comparative linguistics. By virtue of being a single numerical value that is calculated in the same way for any linguistic phenomenon, it can provide researchers with a standard measure of Transitivity that can be used to study the role or effect of Transitivity across constructions both within the same language and across different languages. In addition, the weights associated with each parameter can undoubtedly help us characterise each construction for which Transitivity is thought to be important in much more detail and certainty.

As regards future research directions, like with any statistical model, we should not assume that the models herein are an appropriate representation of speakers’ grammars. These models are a first step to characterise a linguistic phenomenon in a mathematically formal way. A natural next step after proposing a statistical model is to validate its findings from a psycholinguistic perspective by conducting experiments manipulating the parameters for which we have found evidence of an effect. A statistical model alone cannot tell us what speakers do or what features they attend to when using language. Only after finding convergence between human performance and our models can we be more confident that our statistical models may represent the constraints by which speakers operate.

## 5. Conclusion

In this paper I have proposed the Transitivity Index, a weighted continuous measure based on the seminal work by Hopper and Thomson [12]. I have demonstrated its utility and usefulness by analysing the clitic case alternation in causative constructions in Spanish and shown that the Transitivity Index can account for nearly 75% of the alternation. Most importantly, the weighted nature of the index makes it sensitive to the particular construction under study, accounting for the extent to which each parameter may or may not matter in a construction. Therefore, the Transitivity Index shows great promise for comparative linguistics.

Furthermore, the findings in this paper provide evidence that the alternation in clitic case in causative constructions in Spanish can be modelled on the Transitivity parameters proposed by Hopper and Thompson [12]. Model-1 showed evidence for only four of these parameters, namely AgencySubj, AnimacyObj, concreteness and participants and their interactions. However, when the Transitivity parameters were weighted such that their prominence in the construction could be accounted for, the Transitivity Index differentiated between the two clitics almost as well as Model-1, such that the dative clitic was the preferred form for high Transitivity contexts. The results also confirm the traditional account that the dative clitic appears with transitive verbs and the accusative clitic with intransitive verbs but this is a probabilistic, not a categorical, rule.

## Supporting information

S1 Appendix Coefficient estimates, 95% confidence intervals and trace plots. (DOCX)
