# Supplementary material for: The Transitivity Index: Using Transitivity as a continuous measure to account for clitic case alternation in Spanish causative constructions
Source: PLoS One. 2021 Feb 25;16(2):e0246834. doi: 10.1371/journal.pone.0246834 (PMC7906419; doi:10.1371/journal.pone.0246834)
Supplement: S1 Appendix — (DOCX) [file pone.0246834.s001.docx]

S1 Appendix

**Table 1. Posterior mean estimates, 95% credible intervals, Rhat values and effective sample sizes of Model-1.**

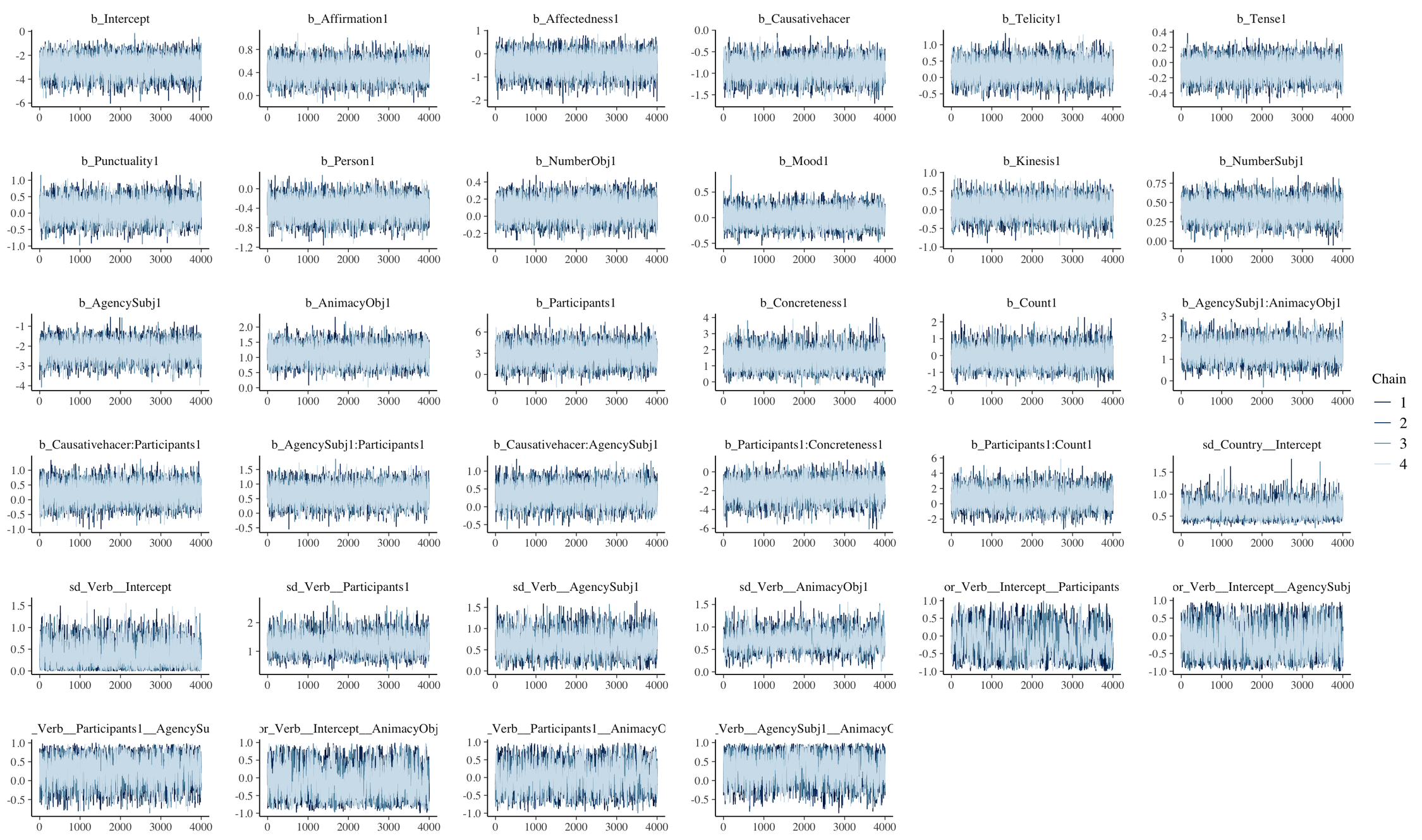


**Fig 1. Trace plots of full Model-1.**

**Table 2. Posterior mean estimates, 95% credible intervals, Rhat values and effective sample sizes of Model-2.**

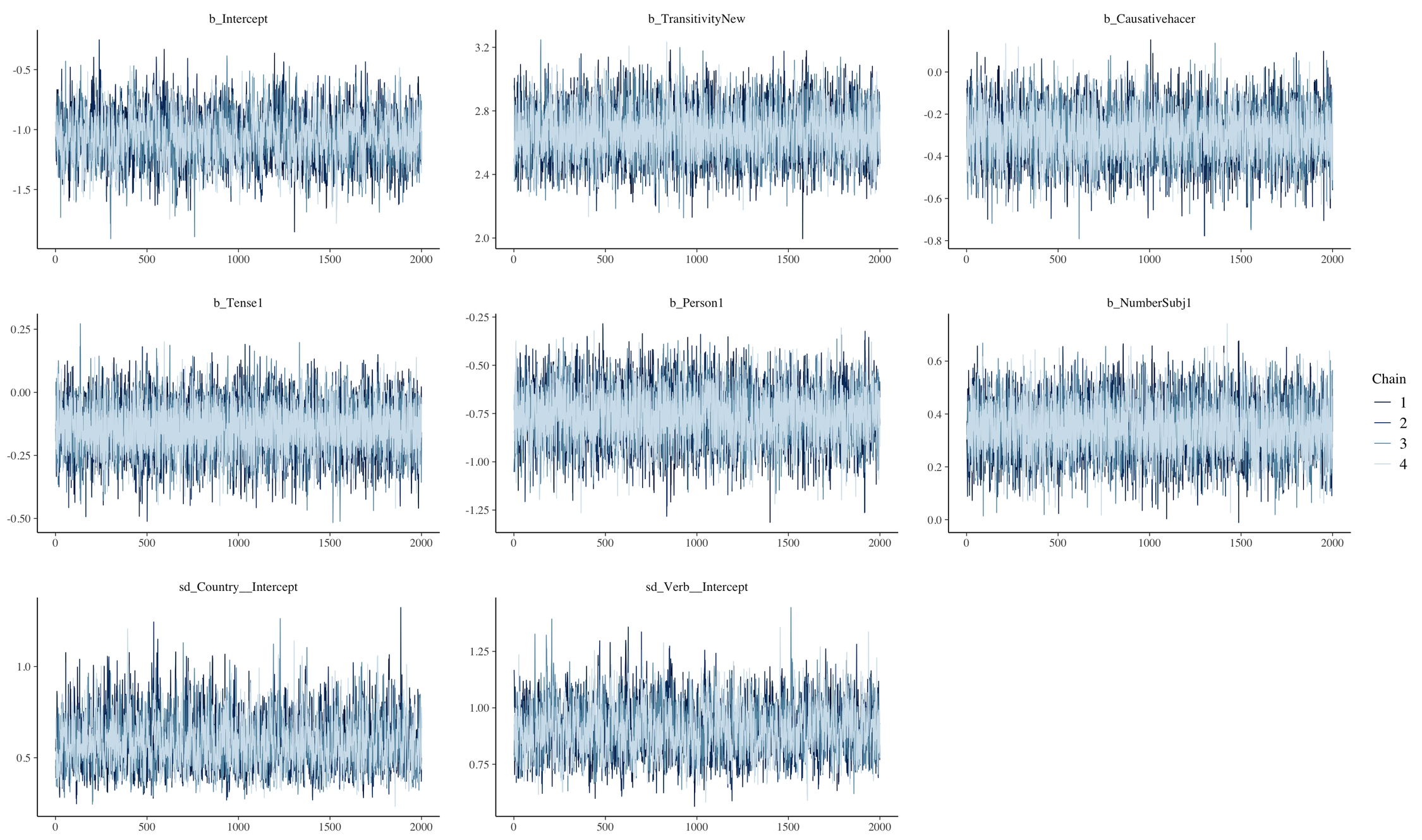


**Fig 2. Trace plots of Model-2.**
